# Individual Differences in Detecting and Correcting Logical Errors in Mathematical Texts

**DOI:** 10.3390/bs16050635

**Published:** 2026-04-23

**Authors:** Zhenhua Luo, Xinyuan Yang, Yong Zhang, Bin Xiong

**Affiliations:** 1 School of Mathematical Sciences, East China Normal University, Shanghai 200241, China; 52215500043@stu.ecnu.edu.cn (Z.L.);; 2 Shanghai Key Laboratory of PMMP, Shanghai 200241, China; 3 School of Mathematics, Yunan Normal University, Kunming 650500, China

**Keywords:** logical errors, mathematical reasoning, error detection and correction, individual differences, think-aloud

## Abstract

This study explored individual differences among senior high school students in detecting and correcting logical errors in mathematical reasoning. Eight participants with high and average mathematical abilities each were recruited from a key high school in Shanghai to solve three error-detecting tasks by thinking aloud; they were then interviewed. Results showed that high ability students performed better in answering time, validation judgment, detection, explanation, and correction of logical errors. The cognitive processes for detecting and correcting logical errors were a combination of five types of cognitive behaviors—read, analyze, check, judge and correct. Although their specific combination methods were different, the two groups exhibited two different detecting styles. Error detection of high ability students was more active and effective, and their thinking processes were smooth and concise. The average ability group was more passive in error detection, more dependent on mathematical texts, and more stuck in the thinking process. Both groups agreed on the value of logical error-detecting tasks, although the high ability group had a more positive attitude toward them.

## 1. Introduction

Logical reasoning is at the core of mathematical abilities, and cultivating and improving students’ deductive reasoning or logical proof ability is an important goal of the high school mathematics curriculum around the world (Ministry of Education of the People’s Republic of China, 2020; Stylianides et al., 2024). Krutetskii (1976) pointed out that “for a mathematician, an important ability is to critically evaluate every aspect of reasoning according to logical principles to identify errors in reasoning that may seem flawless at first glance.” To study the mathematical abilities of primary and secondary school students, Krutetskii used mathematical sophistry questions to analyze their logical reasoning ability, critical thinking, and flexibility. Unfortunately, Krutetskii’s results did not observe the thinking processes and differences between students with different abilities when solving this kind of question. Although it can be expected that students with strong mathematical abilities will perform better in discovering and correcting reasoning errors, a detailed analysis of the thinking process and related influencing factors for students to discover and correct reasoning errors still has important value. It can provide important insights for evaluating and developing students’ logical reasoning and critical thinking, through the use of logical error-detecting tasks.

When there are implicit logical errors in mathematical solutions, can students detect and correct them, and how? What are their views on these problems, and how can they be used in teaching? This study provides empirical data to answer these questions. In short, this study aims to explore the characteristics, and similarities and differences in the cognitive process of detecting and correcting logical errors among high school students with different mathematical abilities, understand learning habits that affect their performance, and understand their views and suggestions on logical error-detecting tasks. The specific research questions were as follows:

1. What are the differences in the performance and cognitive processes between average and high ability students in detecting and correcting logical errors?
2. What are the differences in the mathematical error-detecting experiences and problem-solving habits of average and high ability students?
3. What are the differences between average and high ability students in terms of their views on the value and use of logical error-detecting tasks?

## 2. Literature Review

The literature review consists of two parts: research on error analysis and erroneous examples and research on proof validation. The literature review revealed the connection between this study and the existing research, and the rationale for determining the research tools and analytical frameworks became clear.

### 2.1. Error Analysis and Erroneous Examples

Error analysis mainly explores the nature, types, and causes of errors made by students and is one of the earliest research fields that researchers in mathematics education have paid attention to (Radatz, 1979). With more nuanced error research, the focus has expanded from diagnosis and remediation after making mistakes, to the active utilization of errors to promote student learning (Gao & Xue, 2009). The design and use of erroneous examples is a research topic within the larger idea of learning from errors and has attracted the attention of many researchers. Erroneous examples refer to those that contain incorrect solutions or problem-solving steps (Xu & Zhang, 2011). The error driven learning theory model related to it (Siegler, 2002; VanLehn, 1999) supports students in learning from errors by believing that errors can trigger their reflection and self-explanation, thereby enhancing learning effectiveness. Empirical research has also shown that the use of erroneous examples can effectively promote rule learning in well-structured knowledge domains (e.g., mathematics, physics, and chemistry), when learners have an appropriate knowledge foundation (Booth et al., 2013; Durkin & Rittle-Johnson, 2012; Große & Renkl, 2007; McLaren et al., 2015; M. Wang et al., 2015; Xu & Zhang, 2011; C. Yang et al., 2017; Q. Zhang & Zhang, 2014). The error analysis can also contribute to clarifying the nature of certain types of knowledge and thus to the further development of theoretical perspectives (Dorner et al., 2026).

Mathematical education research on erroneous examples mainly included rule learning in arithmetic, elementary algebra, and probability. The research subjects were mainly primary school students and college students; the main types of errors were conceptual errors and computational errors. There has been little research on logical errors which refer to errors violating logical rules in reasoning and argumentation.

### 2.2. Mathematical Reasoning and Logical Errors

Mathematical reasoning is a special case of reasoning where the process is one of deriving a new mathematical proposition from one or several known mathematical propositions. Reasoning is only correct if it follows the basic laws of formal logic, namely those of identity, contradiction, the excluded middle, and sufficient reason. Mathematical proof is the reasoning process for using a mathematical proposition that has been determined to be true to determine the truth of another mathematical proposition. In this proof, logical errors that are generally unintentional are called fallacies. Intentionally making incorrect arguments is called sophistry. Sophistry aims to give logically untenable arguments a logically correct appearance and pass them off as true. Miyazaki et al. (2017) proposed a theoretical framework that captures students’ understanding of the structure of deductive proofs in terms of three levels of increasing sophistication: pre-structural, partial-structural, and holistic-structural. They contended that accepting logical circularity can be an indicator of lack of understanding of syllogism.

Proof validation refers to the reading which is aimed at checking the correctness of the argumentation process. Previous studies have primarily used expert/novice designs. Selden and Selden (2003) used four proofs of a specific number theory provided by students as research tools to conduct individual interviews with four undergraduate students majoring in mathematics and four undergraduate students majoring in mathematics education. The participants were first asked to attempt to prove the theorem and then presented four proofs in sequence. They were required to decide whether each of them was valid and rethink their earlier decisions with an opportunity to change their minds. The results indicate that undergraduate students tend to focus on the surface features of arguments, and their ability to determine whether arguments are true is limited. Alcock and Weber (2005) used erroneous proof in real analysis as a research tool and asked 13 undergraduate students to determine whether it was a valid proof. Only six undergraduate students believed that the proof was incorrect, and only two of them provided a reasonable explanation for the error. Inglis and Alcock (2012) used eye-tracking technology to record the proof-validation behaviors of 18 undergraduate students and 12 mathematical researchers. The results showed that mathematicians sometimes have divergent judgments on the validity of proofs. Undergraduate students spend more time focusing on the surface features of their arguments and less on the logical structure, while mathematicians tend to shift their attention back and forth between consecutive lines of proof text, devoting more effort to inferring implicit warrants.

Overall, there are relatively few empirical studies on proof validation. These studies focus exclusively on college students and university mathematics teachers, which focus on the cognitive process of proof validation, mainly collecting data through verbal reports and eye-tracking techniques supplemented by interviews. There are certain differences in the results obtained by different researchers (Inglis & Alcock, 2013; Weber & Mejia-Ramos, 2013; Weber, 2008).

## 3. Materials and Methods

### 3.1. Participants

Previous studies on erroneous examples and proof validation have shown that low-achieving learners often find it difficult to detect errors and are easily misled. Considering the confusion caused by logical errors, we chose students with a solid foundation in mathematical knowledge. Sixteen students from a key high school in Shanghai were recruited to participate in the study through purposive sampling. Key high schools are highly regarded as quality high schools within the Chinese education system. They are typically known for their outstanding teaching staff, excellent academic atmosphere, high-level management of education, and outstanding graduation rates. None of the participants experienced any barriers to verbal expression. The high-ability students were eight male students from a science experimental class with excellent math scores of which six won second or third prizes at the National Senior High School Mathematics Competition. As gender differences were not the focus of this study, and the class consisted entirely of male students, no female participants were recruited for the high-ability group. Students with average abilities were from non-science experimental classes, with four males and females each. The participants were coded according to group, sex, and serial numbers. The groups were distinguished by H (high ability) and A (average ability), and sex was indicated by F (female) and M (male). The serial numbers were 01–08 according to the order of participation in the study. For example, HM01 refers to a male student with the number 1 in the high mathematical ability group, and AF01 refers to a female student with the number 1 in the average mathematical ability group. Participants voluntarily joined in this study. Each participant received a ballpoint pen and notebook (worth approximately USD 7) as a token of gratitude.

### 3.2. Materials

Ten alternative tasks were determined, and three tasks for the think-aloud study were determined through student trials, teacher trials, and expert discussions (see Table 1). Appendix A provides the questions and answers. The mathematical knowledge involved in the task is the basic content of junior and senior high school mathematics.

Task 1 (T1) “Prove 5 > 16” is a mathematical sophistry problem (Ma et al., 1981). The error is that ① ($lg\frac{1}{4}>lg\frac{1}{5}$) and ② (2>1) cannot be directly multiplied, because their reasoning premise (a>b, c>d⟹ac>bd) is not valid. This reasoning process is based on false propositions and therefore violates the laws of sufficient reasoning.

Task 2 (T2) “Find the range of the parameter” (Peng, 2007) uses a necessary condition instead of a sufficient and necessary condition to expand the scope of the solution set, which violates the law of identity. $x_{1}>2$, $x_{2}>2$ are sufficient and unnecessary conditions for $x_{1}+x_{2}>4$, $x_{1}x_{2}>4$, and the transformation from the former to the latter is not equivalent.

Task 3 (T3) “Find the minimum value” (F. Wang, 2015) used a basic inequality twice, but the conditions for the equal sign to be true are different in both cases, which violates the law of contradiction. The condition for the equal sign to hold in $1=x+y\geq 2\sqrt{xy}$ is x=y. The condition for the equal sign to hold in $\frac{1}{x}+\frac{2}{y}\geq 2\sqrt{\frac{1}{x}\cdot \frac{2}{y}}$ is that $\frac{1}{x}=\frac{2}{y}$.

### 3.3. Design

The study used a 3 (error type: invalid warrant vs. non-equivalent transformation vs. inconsistent) × 2 (mathematical ability: high ability vs. average ability) design. Among them, error type is an intragroup variable, and mathematical ability is an intergroup variable. The dependent variable was the subject’s error-detecting performance, including problem-solving time, score, and error-detecting behavior.

### 3.4. Procedure

The study was conducted in a quiet self-study room with researchers and individual students. The procedure included four parts: introduction of research objectives, think-aloud exercises, think-aloud studies, and interviews.

Practice material for thinking aloud was adapted from Fox et al. (2011). The researcher first explained to the participants what they assumed think aloud was, then let the participants practice, and decide whether to do more practice based on their performance. The key was to enable participants to synchronously express their thoughts in language while focusing on problem-solving. When students remained silent for more than 15 s, they would be reminded to “continue speaking” and avoid prompts and other questions related to the primary question (Y. Zhang & Xiong, 2021).

After practicing thinking aloud, the participants first completed one warm-up question and then three detecting tasks in sequence without any time limit for answering. After the study, we discussed the correct solution with the participants and answered related questions as compensation.

The interview consisted of two parts: a brief interview (1–5 min) after completing the detecting of each task, with two interview questions aimed at understanding silence, ambiguity, and other thoughts related to the question during each student’s think-aloud process. After completing all tasks, there was a summary interview (10–30 min) with eight interview questions aimed at further understanding the students’ thinking processes, their mathematical learning habits, and their views and suggestions on the use of logical error-detecting tasks (Maher & Sigley, 2014). Appendix B provides an outline of the interviews.

The research process was recorded using a Sogou AI recording pen (C1Pro), and all problem-solving manuscripts were collected after the investigation was completed. The audio recordings of the think-aloud exercise and interviews were translated word by word, and participants were asked to check some of the text to obtain the Think Aloud Protocol (TAPs) and interview texts.

### 3.5. Data Analysis

We calculated the problem-solving time for each task completed by the participants. Each task had a maximum score of 4 points. The following four scoring indicators were used: validation judgment, detection, explanation, and correction of logical errors. A score of 1 was assigned for each indicator that was answered correctly; otherwise, a score of 0 was assigned. Because Task 1 involves a sophistry question, it is obvious that there is an error in the solution, and there was no need to provide a correct solution. Only two indicators—identifying errors and explaining their causes of errors—were rated. If each indicator was answered correctly, a score of 2 was assigned; otherwise, a score of 0 was assigned. Referring to Schoenfeld’s (1985) problem-solving episode and Ye’s (2005) proof-validation process, an analytical framework including five types of error-detecting episodes was formed after repeated discussions and modifications (see Table 2): read, analyze, check, judge, and correct. The two researchers independently encoded all TAPs with a Kappa coefficient of 0.730. Differences were resolved through discussion. Using SPSS 23.0, a repeated-measures ANOVA was performed on the quantitative data from the think-aloud study. The interview data were analyzed using NVivo 12.

## 4. Results

### 4.1. Results of Think-Aloud Study

#### 4.1.1. Problem Solving Time and Task Score

The problem-solving times and scores of high and average ability students in the think-aloud study are shown in Table 3.

The Analysis of Variance (ANOVA) on the problem-solving time showed that the main effect of the level of mathematical ability was not significant (F[1,14] = 3.311, *p* > 0.05); the main effect of the type of logical error was not significant (F[2,13] = 3.158, *p* > 0.05), and there was no significant interaction between the level of mathematical ability and type of logical error (F[2,13] = 0.758, *p* > 0.05).

The analysis of scores showed that the main effect of the level of mathematical ability was significant (F[1,14] = 28.632, *p* < 0.001). High ability students scored significantly higher than average students. The main effect of the type of logical error was not significant (F[2,13] = 0.457, *p* > 0.05). There was no significant interaction between the level of mathematical ability and type of logical error (F[2,13] = 0.160, *p* > 0.05).

The number of participants in the two groups who successfully judged right and wrong, identified errors, explained causes, and wrote the correct solutions is shown in Table 4. In terms of solution validation, all eight high ability students gave correct judgments for Tasks 2 and 3 (Task 1 did not require judgment). The average number of students was five for both groups. Fisher’s exact test showed no significant difference in the number of participants who made correct judgments between the two groups. In terms of identifying errors, all eight high ability students were able to identify the errors. The corresponding numbers for average ability students were two, five, and five, respectively. There was a significant difference in the number of participants who identified errors in Task 1 between the two groups (*p* = 0.007), after the Fisher exact test. In terms of explaining the cause of error, eight high ability students and two average ability students correctly explained the cause of error in T1 (*p* = 0.007, Fisher’s exact test). More high ability students correctly explained the causes of errors in T2 and T3, but there was no significant difference. In terms of writing the correct solution, eight high ability and three average ability students wrote the correct solution at T2 (*p* = 0.026, Fisher’s exact test); eight high ability students and two average ability students provided the correct solution for T3 (*p* = 0.007, Fisher exact test). One average ability student attempted to write the correct solution for T2, and two average ability students attempted to write the correct solution for T3 but were unsuccessful.

#### 4.1.2. Number of Spoken Words and Error-Detecting Episodes

Table 5 shows the number of spoken words and error-detecting episodes in the TAPs for the three tasks. The number of spoken words is obtained by counting the number of Chinese words spoken by students in TAPs.

The ANOVA of the number of spoken words showed that the main effect of the level of mathematical ability was not significant (F[1,14] = 3.182, *p* > 0.05), but the main effect of the type of logical error was significant (F[2,13] = 3.594, *p* < 0.05). The number of spoken words for the non-equivalent transformation task was significantly higher than for the other two tasks, and the interaction between the level of mathematical ability and type of logical error was not significant (F[2,13] = 1.680, *p* > 0.05).

The ANOVA on the number of error-detecting episodes showed that the main effect of level of mathematical ability was not significant (F[1,14] = 0.537, *p* > 0.05); the main effect of type of logical error was not significant (F[2,13] = 0.278, *p* > 0.05); and the interaction between the level of mathematical ability and type of logical error was not significant (F[2,13] = 1.201, *p* > 0.05).

#### 4.1.3. Analysis of the Proportion of Five Types of Error-Detecting Episodes

Table 6 lists the proportions of the five types of error-detecting episodes to the total number of episodes in the three tasks.

The ANOVA on the percentage of reads showed that the main effect of the level of mathematical ability was not significant (F[1,14] = 0156, *p* > 0.05); the main effect of the type of logical error was not significant (F[2,13]) = 2.238, *p* > 0.05); while the interaction between the level of mathematical ability and type of logical error was significant (F[2,13] = 6.445, *p* < 0.05).

The ANOVA on the percentage of analysis showed that the main effect of the level of mathematical ability was not significant (F[1,14] = 0.890, *p* > 0.05); the main effect of the type of logical error was not significant (F[2,13] = 2.920, *p* > 0.05); and the interaction between the level of mathematical ability and type of logical error was not significant (F[2,13] = 2.856, *p* > 0.05).

The ANOVA on the percentage of checks showed that the main effect of the level of mathematical ability was not significant (F[1,14] = 2.097, *p* > 0.05); but the main effect of the type of logical error was significant (F[2,13] = 5.157, *p* < 0.05).

The multiple comparison results of the average ability students showed that the percentage of checks in the invalid warrant task was significantly higher than that in the non-equivalent transformation task, the percentage of checks in the inconsistent task was significantly higher than that in the non-equivalent transformation task, while there was no significant difference in the percentage of checks between the invalid and inconsistent tasks. There was no significant difference in the percentage of checks among the three task types for high ability students. The interaction between the level of mathematical ability and type of logical error was significant (F[2,13] = 5.737, *p* < 0.05).

The analysis of the percentage of judgments showed that the main effect of the level of mathematical ability was not significant (F[1,14] = 0.675, *p* > 0.05); while the main effect of the type of logical error was significant (F[2,13] = 3.387, *p* < 0.05). The multiple comparison results of the average ability students showed that the percentage of judgments in the invalid warrant task was significantly higher than in the non-equivalent transformation task, the percentage of judgments in the inconsistent task was significantly higher than in the non-equivalent transformation task, while there was no significant difference in the percentage of checks between the invalid and inconsistent tasks. The multiple comparison results of high ability students showed that the percentage of judgments in the invalid warrant task was significantly higher than in the non-equivalent transformation task, the percentage of judgments in the inconsistent task was significantly higher than in the non-equivalent transformation task, but there was no significant difference in the percentage of checks between invalid and inconsistent tasks. The interaction between the level of mathematical ability and type of logical error was not significant (F[2,13] = 2.960, *p* > 0.05).

The ANOVA on the percentage of correct answers showed that the level of mathematical ability was not significant (F[1,14] = 2.437, *p* > 0.05), while the main effect of the type of logical error was significant (F[2,13] = 43.141, *p* < 0.001). The multiple comparison results for average ability students showed no significant difference in the percentage of correct answers among the three tasks. The multiple comparison results for high ability students showed that the percentage of correct answers in the non-equivalent transformation task was significantly higher than that in the invalid warrant task; there was no significant difference in the percentage of correct answers between the non-equivalent transformation and inconsistent tasks, and there was no significant difference in the percentage of correct answers between the inconsistent and invalid warrant tasks. The interaction between the level of mathematical ability and type of logical error was not significant (F[2,13] = 3.230, *p* > 0.05).

#### 4.1.4. Case Analysis of TAPs

From the TAPs of the students, it can be observed that there are two types of error-detection patterns (the proactive error-detection pattern and the passive error-detection pattern). A qualitative analysis of the TAPs of both sets of students showed that there are two common patterns of error-detecting: (1) reading the problem and solution line-by-line, detecting the errors at the first reading, then explaining the reasons for the errors, and providing the correct solution; (2) if errors cannot be directly discovered after reading the problem and its solution, high ability students usually tried to write the correct solution, and then compare it with the provided one to detect the errors and explain the reasons. In contrast, the performance of average ability students was significantly inferior, and in most cases, errors were detected through repeated reading, which could be easily distracted by an incorrect solution. The following is a case analysis of two participants’ performances on Task 3, where the above differences can be seen.

HM01 skipped the problem and solution and immediately said to “use Cauchy’s inequality to calculate it first.” After obtaining the correct solution, he compared it with the provided solution to identify and explain the errors. HM01 used 75 s to complete the task. His detecting routes were R1 → A1 → Ch1 → G2 → Ch1 → G2. The TAPs are as follows.

1. x>0*,* x+y=1*,* $\frac{1}{x}+\frac{2}{y}$.

2. *Use Cauchy’s inequality to calculate it first, I will solve it first and then see if it is correct.*

3. *Cauchy, um …. …[pause for 11 s, write down* $(x+y)(\frac{1}{x}+\frac{2}{y})\geq {\left(1+\sqrt{2}\right)}^{2}$*]*

4. *It is wrong.*

5. *Oh, let me take a look.*

6. *It is because* x+y=1*, so* $1=x+y\geq 2\sqrt{xy}$*,* $xy\leq \frac{1}{4}$ *… [pause for 5 s]*

7. *It is incorrect, because the equality conditions of these two equations do not match.*

8. *In the second step, it is said that* x+y *is smaller, greater than or equal to* $2\sqrt{xy}$*. In this case, the equality condition is* $x=y=\frac{1}{2}$.

9. *But in the following step,* $\frac{1}{x}+\frac{2}{y}\geq 2\sqrt{\frac{2}{xy}}$*, its equality condition is different from that,* x=y.

10. *But in the subsequent step, it directly substituted the results of* x equals y.

11. *So, this is wrong.*

During the TAPs of AM04, a simple analytical behavior was observed, followed by a line-by-line reading and checking of the solution step. However, the checking mainly focused on whether each calculation step was correct and did not mention the conditions for the equal sign to hold. After checking the entire solution step, AM04 felt that there were no problems, but also expressed doubt and uncertainty. He said, “I always feel strange” and briefly remained silent. However, this did not lead to errors in detection. AM04 then conducted another calculation check for the solution, and after confirming that there were no calculation errors, he believed that the solution was correct. His problem-solving time was 345 s, and the detecting route was R1 → A1 → Ch1 → L1 → Ch1 → L1 → G3 → Ch1 → G2.

### 4.2. Results of Interviews

The results of the interviews are presented in two parts: participants’ error-detecting experiences and problem-solving habits and participants’ perceptions of the value of mathematical logical error-detecting tasks and their use.

#### 4.2.1. Participants’ Error-Detecting Experience and Problem-Solving Habits

Both groups of participants had experience of discovering errors in mathematical solutions in their daily studies. The errors found by average ability students were mainly computational and typographical, while logical errors were rare. Solution errors found by high ability students covered teaching accessory materials, regular exams, and even large-scale exams. There was a significant difference between the two groups in the number of people who found the problem wrong (*p* = 0.001, Fisher’s two-sided accuracy test). Only one average ability student (AF07) found that a problem itself was wrong, and her answer was “It seems that there was one, but then the teacher did not care about it, and then the classmates did not seem to pursue it, and that is it.” Students’ impressions of the problem were vague and difficult to accurately describe. However, all eight high ability students stated that they had identified flawed problems and could accurately recall an impressive example.

There was a significant difference between the two groups in terms of whether they had a habit of solving one problem using multiple solutions (*p* = 0.001, Fisher’s two-sided exact test). Only one student from the average ability group answered that he sometimes solved a problem using multiple solutions, while the remaining seven students clearly stated that they usually did not try to solve problems using multiple solutions. The main reason for this was laziness in learning and thinking. It is good to be able to get the answer according to the most familiar and common ideas when solving math problems. Insufficient mathematical ability and the difficulty of high school mathematics are already high for them, making it difficult to master regular mathematical content. All eight students in the high ability group answered that they would try to solve a problem using multiple solutions. These students tend to seek concise and effective solutions and evaluate the simplicity and complexity of their own solutions in the hope of better understanding the problem through different solutions. Typical responses from the two groups of participants were as follows:

*AM06: Trying different methods to solve a problem is something I usually do not do, unless the teacher asks me to think of another method, or the question directly asks for it. If it does not require me to solve problems using other solutions, and I am sure I will just use the one that comes to mind as the easiest way to solve it.*

*HM06: This is true. It is typical to solve relatively easy problems using multiple solutions. If you encounter difficult problems, for example, you first solve them with a method, and then feel that it is particularly complicated. …… At this point, we should consider whether this solution can be simplified. Is this easier?*

There was a significant difference between the two groups in their habits of checking and reflecting on the solution (usually, sometimes, usually not) (*p* = 0.038, Fisher’s two-sided exact test). Four average ability students responded that they usually checked and reflected on the problem solution with the main purpose of avoiding mistakes, while the other four responded that they usually did not check or reflect on the problem solution. In contrast, five high ability students answered that they often do it, while three students answered that they sometimes do it. High ability students’ purpose for checking and reflecting is to avoid making mistakes, reflect on whether there are any redundancies or reversals in the solution, and seek better solutions.

#### 4.2.2. Participants’ Reflections on and Perceptions of the Use of Logical Error-Detecting Tasks

All participants recognized the value of logical error-detecting tasks in mathematics, and the main values included cultivating rigorous and critical thinking, developing new ideas and opening up the mind, reminding attention, deepening impression, improving understanding, and avoiding the recurrence of similar mistakes. They also recognized the need to improve one’s ability to find and correct one’s own mistakes in regular study and exams, one’s ability to find and correct the mistakes of classmates, one’s reading comprehension and proof validation abilities, and being useful for teachers’ teaching and problem proposing. Adding logical errors to solutions for students to detect and correct can make mathematics reading comprehension involve significant elements of problem solving. Typical answers were as follows:

*AF02: I think it is very valuable because I usually make mistakes and I may have made similar logical mistakes. However, at that time, I could not determine what was wrong. Looking for my mistakes was a bothersome process. You performed the mathematical problem for a long time, thought your solution was very reasonable, but the result was wrong, and then it would be very annoying and irritable. …… You emphasizing to me over and over again is not really helpful; I forget after a while, and you asked me to detect and correct mistakes, like this, is still quite useful.*

*HM07: The solution process itself also has a framework. For example, this part of the solution is about this, and another part of the solution is about that. You should always check the whole topic and obtain a general idea of the logic of the solution, first calculating this step, then putting this step, and then through these two conditions. To detect logical errors, one should know the whole topic and the big picture of the solution.*

Both groups agreed that logical error-detecting tasks could be used in classroom teaching and exercises. However, the two groups did not agree on whether they could be used in the examinations. All eight high ability students indicated that they could be used for the examinations. Two average ability students explicitly disagreed with their use in the exam.

*AM06: I feel that if these questions just look at the difficulty of the problem itself, it may be a moderately difficult or simple problem. This gives an incorrect solution and allows us to find the error from the solution. Sometimes, I feel that it is quite difficult; that is, I know it is wrong, but I cannot find where it is wrong.*

*AM04: I do not think it is a good idea to put this kind of problem in the exam because no one is willing to do this kind of thing in the exam, right? If I were to do this kind of problem in the exam, I would probably put it at the end. If I might be done with the last problem or find that I just cannot do the last problem, I think I might just go for this kind of problem. This type of problem is time consuming.*

## 5. Discussion

This study asked high school students with high and average mathematical abilities to solve three logical error-detecting tasks using a think-aloud method. The cognitive process of detecting and correcting logical errors included five types of cognitive episodes: read, analyze, check, judge, and correct. Owing to differences in the task difficulty, task characteristics, and subject characteristics, the combination of the five types of episodes varied. Although the specific form of combination may be different, the two groups of participants exhibited two different detecting styles. High ability students were more proactive and effective in detecting, and their thinking process was smooth and concise. Average ability students were relatively passive, relied more on the solution text, and had more lag in the thinking process.

There is no significant difference in the number of high or average ability students who have had the experience of discovering incorrect solutions in their studies. However, there is a significant difference in those who have had the experience of discovering flawed problems and in the number of students who had the habit of solving one problem using multiple solutions and of checking and reflecting on solutions. All the participants recognized the value of logical error-detecting tasks, which mainly included exercising thinking, deepening impressions, enhancing understanding, cultivating reading comprehension and error correction abilities, and being useful to mathematics teachers in teaching and problem propositions. Participants supported the use of mathematical error-detecting tasks in classroom teaching and exercises. High ability students had comparatively more positive attitudes toward mathematical logical error-detecting tasks.

Table 3 shows that the time taken by high ability students to complete tasks was less than that of average ability students, but there was no significant difference. This is partly due to the fact that high ability students have gone through a complete cognitive process of identifying, explaining, and correcting errors, while average ability students have the following reasons for ending tasks early: terminating tasks without discovering logical errors; discovering logical errors but terminating the task due to the inability to reasonably explain them; and forgetting or being unable to write the correct solution after identifying and explaining errors. Based on Table 5, it can be seen that high ability students spoke more words in less time and with slightly fewer episodes than average ability students. Thus, it can be inferred that high ability students have a smoother and more organized problem-solving process, whereas average ability students experience a larger lag in their thinking processes. Repeated listening to the audio recordings confirmed these findings.

There were no significant differences in the percentages of read, analyze, check, judge, and correct answers between the two groups. The number of participants in both groups is relatively small, which may lead to insignificant differences. Additionally, the reason for the insignificant differences is likely that the abilities of both groups of participants are above the medium level, with one group being excellent and the other being average. The reason for not selecting students with poor mathematical foundation is that the existing research on erroneous examples indicates that students with poor foundation tend to get confused and lost when facing errors (Dieterich et al., 2025). The results in Table 6 indicate that there was no significant difference in the percentage of correct answers for invalid and inconsistent tasks among high ability students, mainly due to the inclusion of alternative solutions in the check episode. Three high ability students did not consider the provided solutions. Instead, they provided their own solutions and compared them with existing ones. Actively attempting and successfully completing their own solutions was an important difference between the two groups of participants.

The data in Table 4 indicate a decreasing trend in the number of students who made correct judgments, successfully identified errors, explained the causes of errors, and wrote the correct solutions. This indicates that the correct judgment of the solution does not necessarily guarantee the detection of a specific error location. The judgment of the solution is related to the sense of the question and the examination requirements (no one chooses uncertainty because the examination does not allow it) and has a certain degree of probability. Being able to identify the location of a logical error does not necessarily mean being able to clearly explain the cause of the error. Similarly, being able to explain the reason for an error does not necessarily guarantee that the correct solution can be written. For average ability students, it may be more effective to approach incorrect solutions based on a clear understanding of the correct solutions. AF01 performed the worst among all participants and failed to identify any errors when completing the three tasks. In the interview section on the use of logical error-detecting tasks, the dialogue was as follows:

*AF01: I think if you just come across a problem and an erroneous solution and are asked to read it. I think it is not good; it is definitely not good. I think, even if you do not let your classmates do it first, you should show them the right solution first and then let them understand the right solution. You can then read the wrong one and tell them exactly where it was wrong. This is more effective.*

*Researcher: Present the correct solution first, and then build upon it…*

*AF01: Yes, I have also found that as long as you see for the first time that it is wrong, you may go in the wrong direction. First, it is best to determine what is correct. The first impression in the brain should still be correct, and wrong impressions should not appear first. When you know that it is right and then look at the wrong one, you will feel that it is incorrect, which will lead to better learning. You present an erroneous solution first, then I think for a while, and then I realize that I do not even know where it went wrong, and I think, oh, quite right. I will definitely do so the next time I see this.*

**Figure 1:**
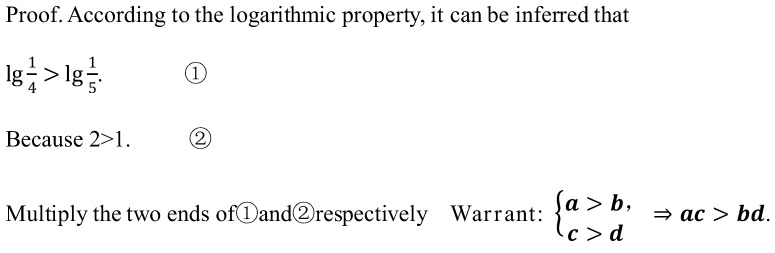
The form of task design to correct the invalid warrant.

When examining an argument, readers face a triple task (Inglis & Alcock, 2012): deciding whether a warrant is required; inferring an appropriate warrant; and evaluating the inferred warrant. For students with insufficient reasoning ability, the following measures can be considered: directly revealing the incorrect warrant with strong concealment and explaining as clearly as possible why the warrant is incorrect. As students improve their abilities, the prompts can be gradually removed, increasing their difficulty in explaining errors. Taking the mathematical sophistry question “Prove 5 > 16” as an example, to focus students’ attention on the warrant, the task can be alternately posed as follows: A student’s problem-solving solution involves the following steps (Figure 1). The student multiplies the two ends of inequalities ① and ②, respectively, and provides the warrant, but this warrant is invalid. Please explain what was wrong with this:

By embedding logical errors, the solution validation involves certain problem-solving elements and is challenging to some extent. The better problem-solving performance of high ability students is expected to lead to their better performance in the detection and correction of logical errors. The interview results on the learning experience and problem-solving habits related to errors among the participants showed significant differences between high and average ability students in their habits of solving one problem using multiple solutions and of checking and reflecting on the solution. High ability students have the habit of solving one problem using multiple solutions and are willing to seek beautiful and concise solutions. The check and reflection on the problem-solving solution not only avoid mistakes but also focus on optimizing the problem-solving solution, which represents the manifestation of mathematical flexibility (X. Yang et al., 2025). The vast majority of average ability students do not have the habit of solving one problem using multiple solutions and the willingness and ability to solve one problem using multiple solutions. When solving problems, they are satisfied with using only conventional methods to obtain answers. Half the students in the average ability group have the habit of checking and reflecting on problem-solving solutions, and their main purpose for this is to avoid errors. The interview results on the value and use of logical error-detecting tasks showed that high ability students had a more positive attitude toward errors, a deeper understanding of their value, and a more open attitude toward their use in various learning and assessment scenarios. Cultivating students’ positive beliefs about using errors as learning resources may help improve their enthusiasm for using errors and cultivate the habit of solving one problem using multiple solutions and checking and reflecting on solutions.

## 6. Conclusions

This study explored the individual differences in detecting and correcting logical errors in mathematical texts with the think-aloud method. The analysis of cognitive processes of subjects presented two different error-detecting styles. High ability students are more active in finding and fixing logical errors, while average ability students are relatively passive and more dependent on the solution text. Specifically speaking, the majority of average ability students directly read the solutions after reading the questions and are easily led by the solutions. Otherwise, the majority of high ability students usually think independently and try to write their own solutions after reading the questions. Why does this difference occur? The interview results showed that the differences between the two groups are related to their daily error-detecting experience and learning habits. There are significant differences in the number of students who have the habit of solving one problem with multiple solutions and active reflection on the given solutions. Math teachers can use multi-solution problems to encourage multiple approaches and help students compare solutions—including flawed ones. Existing studies on multiple-solution tasks are usually related to mathematical creativity (Gridos et al., 2021; X. Yang et al., 2025), Exploring the relationship between multiple-solution tasks, mathematical creativity and students’ error-detecting ability is an interesting topic.

The logical error-detecting tasks used in this study are a specific type of erroneous examples. A systematic review of learning from erroneous examples examined how influential factors affect the effectiveness of erroneous examples (Dieterich et al., 2025). This review concentrates on five influential factors, namely prompts, feedback, error context, cognitive load, and prior knowledge. The results show that the benefits of erroneous examples depend on how errors are highlighted, how prompts are designed, and on learners’ prior knowledge and cognitive abilities. Given the fact that the evidence from some existing studies appears to be contradictory, further empirical research is needed to determine how these factors must be balanced to clarify under which conditions erroneous examples are most effective (Barbieri et al., 2023; Kopp et al., 2008; Loibl & Leuders, 2019). When using logical error-detecting tasks in mathematics learning and teaching, we also suggest taking all above five factors into consideration. In addition, our results showed that the high ability students had a more positive attitude toward erroneous examples. Focusing on the effective factors of students’ use of erroneous examples, creating a supportive learning environment that encourages productive error-making, and helping students develop a positive attitude toward errors can make erroneous examples more effective.

## 7. Limitations

This study had several limitations. This study used convenience and purposive sampling, with only 16 senior students from the same key high school in Shanghai. The research conclusions are more representative of the average number of high ability students. To explore the cognitive processes of error detection and correction, this study used the think-aloud method, which makes it difficult to capture automated and unconscious thinking processes (such as epiphany). Therefore, the participants’ verbal reports may not be complete. Eye-tracking technology can be used to measure the allocation and transfer of attention when subjects read mathematical text, as a supplement to think-aloud data. The types of logical errors involved in this study included invalid errors, non-equivalent errors, and inconsistencies. Each error type corresponds to only one task, and the attributes of these tasks, other than the error type, may have also affected the participants’ performance in detecting. The mathematical content of the tasks is mainly limited to algebraic knowledge. Therefore, future research should further increase the number of tasks and involve other types of logical errors (such as circular argumentation) and mathematical fields (such as geometry, calculus and number theory).

## Figures and Tables

**Table 1:** Mathematical knowledge and reasons for logical error-detecting tasks.

Task	Mathematical Knowledge	Reason for Error
Prove 5 > 16	Property of inequality	Invalid warrant
Find the range of the parameter	Necessary and sufficient condition	Non-equivalent transformation
Find the minimum value	Basic inequality	Inconsistent

**Table 2:** Analysis framework for the cognitive process of error-detecting.

Episode	Definition
Read	Reading the question and solution, silence and rereading. Including (R1) First Reading: The participants read part or all of the detecting text for the first time. (R2) Re-reading: The participants re-read part or all of the error-checking text.
Analyze	Analyze the text to understand its meaning, accompanied by the recall and activation of existing knowledge. Including (A1) Analysis of condition: Clarify the condition and the unknown, present condition in the form of a chart, analyze the meaning of the question, and actively recall relevant knowledge or solutions. (A2) Analysis of the solution: Comment on the overall impression of the solution, identify its pattern, divide the solution into several parts, clarify the relationship between the solution steps, and identify the warrant of a certain step.
Check	Check and verify whether the solution steps are correct, providing a basis for making judgments. Including (Ch1) Alternative solution comparison: Try to provide the solution independently. (Ch2) Calculation check: Check for errors in the calculation process using pen or mental calculation. (Ch3) Inference check: Check whether the inference is valid, identify the premise of reasoning, and verify whether the premise is valid. (Ch4) Example: Construct counterexamples or special case validations. (Ch5) Comparison with principle: Explain the reasons for errors by recalling correct mathematical facts. (Ch6) Combining numbers and shapes: Draw figures to verify.
Judge	Judge the correctness of solution, including (J1) Partial judgment on the solution steps: believe that a certain step is correct; believe that a certain step is incorrect; express doubt or uncertainty. (J2) Overall judgment on the solution: believe that the solution is correct or only requires minor modifications; believe that the solution is incorrect; express doubt or uncertainty.
Correct	The process of correcting solution, including (Co1) Partial correction (Co2) Rewriting the solution: The examination of the participants’ self-correction process is also coded as Correct.

**Table 3:** Behavioral data of high ability and average ability students in think-aloud study: problem-solving time and scores (M ± SD).

	Invalid Warrant		Non-Equivalent Transformation		Inconsistent	
	Time (s)	Score	Time (s)	Score	Time (s)	Score
Average Ability	261.88 ± 135.21	2.13 ± 1.55	304.13 ± 196.86	1.63 ± 1.60	346.13 ± 182.15	1.88 ± 1.81
High Ability	119.50 ± 42.49	4.00 ± 0.00	289.88 ± 137.81	3.88 ± 0.35	214.13 ± 126.52	3.88 ± 0.35

**Table 4:** Number of participants in the two groups who successfully judged right and wrong, identified mistakes, explained causes and wrote the right solutions.

	Invalid Warrant		Non-Equivalent Transformation		Inconsistent	
	Average Ability	High Ability	Average Ability	High Ability	Average Ability	High Ability
Correct judgments	*	*	5	8	5	8
Identified mistakes	2	8	5	8	5	8
Explained causes	2	8	4	8	4	8
Wrote right solutions	*	*	3	8	2	8

* Task 1 is a sophistry question. There was no need to provide a correct solution.

**Table 5:** Number of spoken words and error-detecting episodes (M ± SD).

	Invalid Warrant		Non-Equivalent Transformation		Inconsistent	
	Spoken Words	Episodes	Spoken Words	Episodes	Spoken Words	Episodes
Average Ability	251.13 ± 149.87	9.63 ± 4.47	289.25 ± 148.73	8.88 ± 4.67	255.88 ± 95.42	11.00 ± 5.43
High Ability	242.75 ± 125.66	8.50 ± 1.93	494.50 ± 252.05	9.25 ± 2.71	361.25 ± 194.38	8.38 ± 2.07

**Table 6:** Percentage of five types of error-detecting episodes (M ± SD).

	Invalid Warrant		Non-Equivalent Transformation		Inconsistent	
	Average Ability	High Ability	Average Ability	High Ability	Average Ability	High Ability
Read	0.192 ± 0.08	0.297 ± 0.063	0.227 ± 0.105	0.163 ± 0.058	0.234 ± 0.089	0.227 ± 0.069
Analyze	0.016 ± 0.030	0.081 ± 0.075	0.135 ± 0.132	0.088 ± 0.104	0.026 ± 0.036	0.096 ± 0.111
Check	0.445 ± 0.128	0.280 ± 0.115	0.255 ± 0.137	0.328 ± 0.139	0.453 ± 0.077	0.360 ± 0.104
Judge	0.318 ± 0.097	0.342 ± 0.053	0.032 ± 0.071	0.128 ± 0.067	0.351 ± 0.105	0.292 ± 0.110
Correct	0.030 ± 0.044	0.000 ± 0.000	0.032 ± 0.071	0.128 ± 0.067	0.047 ± 0.074	0.078 ± 0.123

## Data Availability

The raw data of this article will be made available by the authors, without undue reservation.

## Appendix A. Logical Error-Detecting Tasks

1. Read the following proof.
  **Proof.** 
  **(5 > 16)**
  According to the logarithmic property, it can be inferred that
  $lg\frac{1}{4}>lg\frac{1}{5}$. ①
  Because 2>1. ②
  Multiply the two ends of ① and ② separately yields
  $2lg\frac{1}{4}>lg\frac{1}{5}$, Namely, $lg{\left(\frac{1}{4}\right)}^{2}>lg\frac{1}{5},lg\frac{1}{16}>lg\frac{1}{5}$. So $\frac{1}{16}>\frac{1}{5},$ that is, 5 > 16. □

Answer the question after reading: Explain why the above proof leads to the absurd conclusion of “5 > 16.”

2. Read the following problem and solution.

If both roots of equation $x^{2}+(m-2)x+5-m=0$ are greater than 2, find the range of values for the real number m.

Solution. If the roots of the equation are $x_{1}{,x}_{2}$, then there are $x_{1}>2$, $x_{2}>2$.

According to the relationship between roots and coefficients, it can be inferred that

$x_{1}{+x}_{2}=2-m,x_{1}x_{2}=5-m$.

So there are $2-m=x_{1}{+x}_{2}>4$, $5-m=x_{1}x_{2}>4$.

Due to the existence of two roots, it can be inferred that

${∆=(m-2)}^{2}-4\times (5-m)=m^{2}-16\geq 0$.

Solve $\left\{\begin{array}{c} m^{2}-16\geq 0,\\ 2-m>4,\\ 5-m>4, \end{array}\right.$ it can be inferred that $\left\{\begin{array}{c} m\leq -4\;or\;m\geq -4,\\ m<-2,\\ m<1. \end{array}\right.$

So, m≤−4.

Answer the following question after reading: Is the above solution correct? If incorrect, point out the location of the error (marked with a line), explain the reason for the error, and write the correct solution.

Answer: □ Correct □ Incorrect □ Unknown □ Other ( )

3. Read the following problem and solution.

Given that x>0, y>0, and  x+y=1, find the minimum value of $\frac{1}{x}+\frac{2}{y}$.

Solution: Because x>0, y>0, and x+y=1,

So $1=x+y\geq 2\sqrt{xy}$, $xy\leq \frac{1}{4}$, $\frac{1}{xy}\geq 4$.

and $\frac{1}{x}+\frac{2}{y}\geq 2\sqrt{\frac{1}{x}\cdot \frac{2}{y}}=2\sqrt{\frac{2}{xy}}\geq 2\sqrt{2\times 4}=4\sqrt{2}$.

Thence the minimum value of $\frac{1}{x}+\frac{2}{y}$ is $\;4\sqrt{2}$.

Answer the following question after reading: Is the above solution correct? If incorrect, point out the location of the error (marked with a line), explain the reason for the error, and write the correct solution.

Answer: □ Correct □ Incorrect □ Unknown □ Other ( )

## Appendix B. Interview Outline

Interview questions after completing each task:

1. Regarding the silence, unclear listening, or ambiguous meaning in the think aloud, ask the participants their thoughts at the time.
2. Do you have any other ideas that need to be added to the task you just completed?

Interview questions after completing all three tasks:

1. What are the similarities and differences between the logical error detecting tasks just completed and the mathematical problems you usually encounter?
2. How do you determine whether an solution is correct or incorrect?
3. When you try to determine whether a solution is valid, do you check every step of the solution?
4. Do you repeatedly read the problem or solution?
5. Have you ever had the experience of discovering errors in the solution or flaws of the problem itself in your daily math learning? If so, please provide a brief description.
6. Do you try to solve one problem using multiple solutions? Do you try to check and reflect on the solution?
7. Do you think that a logical error-detecting task is valuable for mathematics learning? If so, what is the value? If not, why? Do you have any other ideas or suggestions for logical error detecting tasks or their use?
8. Has it caused you a mental burden to solve problems by asking you to think aloud? Will it change your normal thinking process?
